# Partially Penetrant Postnatal Lethality of an Epithelial Specific MicroRNA in a Mouse Knockout

**DOI:** 10.1371/journal.pone.0076634

**Published:** 2013-10-07

**Authors:** D’Juan T. Farmer, Nikki Shariat, Chong Yon Park, Huey Jiin Liu, Anastasia Mavropoulos, Michael T. McManus

**Affiliations:** 1 Department of Microbiology and Immunology, University of California San Francisco, San Francisco, California, United States of America; 2 Department of Medicine, University of California San Francisco, San Francisco, California, United States of America; 3 UCSF Diabetes Center, University of California San Francisco, San Francisco, California, United States of America; 4 WM Keck Center for Noncoding RNAs, University of California San Francisco, San Francisco, California, United States of America; University of Texas, MD Anderson Cancer Center, United States of America

## Abstract

MicroRNAs are small noncoding RNAs thought to have pivotal roles in numerous diseases and developmental processes. However, a growing body of literature indicates that *in vivo* elimination of these tiny RNAs usually has little to no observable consequence, suggesting functional redundancy with other microRNAs or cellular pathways. We provide an in-depth analysis of miR-205 expression and define miR-205 as an epithelial-specific microRNA, and for the first time show that ablation of this microRNA knockout exhibits partially penetrant lethality in a constitutive mouse knockout model. Given the role of this microRNA in cancer and development, this mouse model will be an incredible reagent to study the function and mechanisms of miR-205 in epithelial tissue development and disease.

## Introduction

Noncoding RNAs are now recognized as key components of gene regulation. While the classes of noncoding RNAs are a growing list, microRNAs (miRNAs) are among the most characterized. miRNAs are small, ∼22 nt RNAs that function to negatively regulate gene expression by targeting complementary mRNAs [1]. These non-coding regulatory RNAs have been hypothesized to have pivotal roles in numerous diseases and developmental processes and to date, thousands of miRNAs have been identified [2], [3], [4], [5]. While implicated in a variety of biological processes, only a small subset of miRNAs has been demonstrated to be essential [6]. Instead, a growing body of literature suggests that elimination of both single miRNAs and miRNA families has little to no observable consequence in unstressed conditions [7], [8]. This observation is shifting the framework of how researchers consider miRNA biology in human development and disease. However, despite thousands of published studies on miRNA function in cell culture systems, most miRNAs have yet to be studied *in vivo*, leaving a vast gap in the understanding of miRNA-mediated regulation.

Extensive miRNA profiling has given a low-resolution foundation for when and where miRNAs are expressed in several vertebrate models [9], [10], [11]. MiR-205, an intergenic miRNA, is abundantly expressed in the skin of E17.5 mice and has been detected in footpad epithelium, tongue, epidermis, and corneal epithelium, suggesting that it may be a stratified squamous epithelial specific miRNA [12], [13]. Stratified squamous epithelium is composed of squamous epithelial cells that overlay a single layer of epithelial cells that cover a basement membrane. This epithelium has two types, a keratinized type, where the keratin functions to protect surfaces from abrasion, and a non-keratinized type, where hydration is dependent on external secretions. In humans, disruption of epithelial layers can have detrimental consequences, leading to a number of pathologies, making miR-205 an interesting miRNA for further evaluation. Moreover, miR-205 has been shown to target *ZEB1* and *ZEB2*, mediators of epithelial to mesenchymal transition (EMT), in order to maintain an epithelial state [14], [15]. In addition, several cell culture based reports have revealed a role of miR-205 in targeting several regulators of proliferation [16], [17], [18]. While little *in vivo* data is available for miR-205, the tumor protein p63 has been found to regulate its expression and is also essential for mouse development and proper squamous epithelium differentiation and maintenance [19], [20]. Given this growing body of literature, we sought to generate a miR-205 knockout (KO) to fully ascertain the expression profile of this miRNA and to develop a better understanding of its biological significance *in vivo*.

In this study, the expression of miR-205 and constitutive KO phenotypes of a miR-205 mouse line was characterized. miR-205 exhibited striking temporal and spatial epithelial expression patterns during embryonic development. Although KO animals were born normal, they exhibited a weight decrease by postnatal day 7 and a partially penetrant postnatal lethality by postnatal day 14. These data reveal an important role for miR-205 in stratified squamous epithelial-derived tissues, providing an *in vivo* model for further understanding the roles of a noncoding RNA in basic physiology and potentially in the context of human disease.

## Results and Discussion

### miR-205 exhibits temporal and spatially distinct embryonic expression

**Figure 1:**
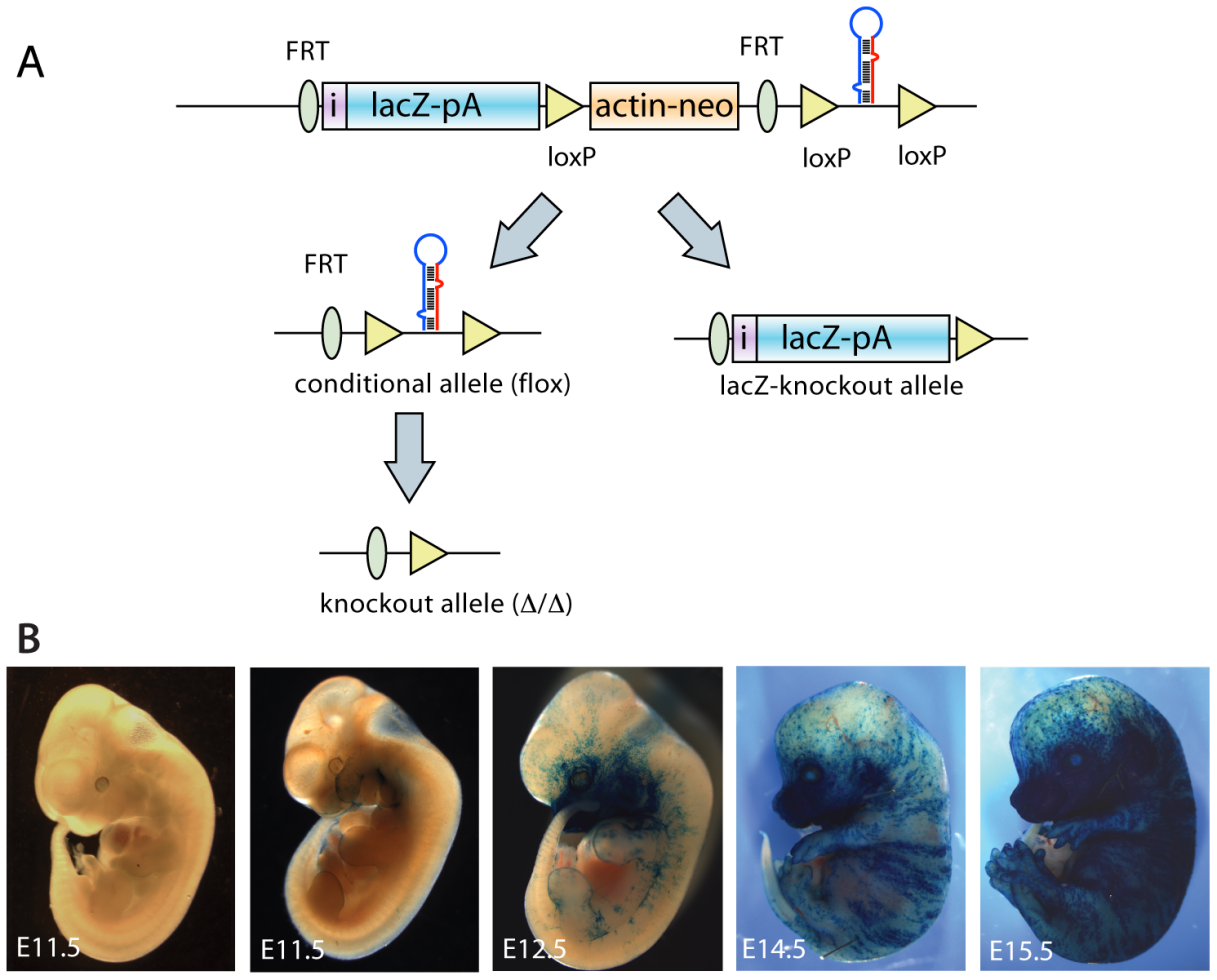
A targeted miR-205-lacZ reporter is functional in mouse embryos. A. Two targeting strategies were used for miR-205 analyses. All lacZ stained embryos were generated in a mixed background crossed directly to Actin-Cre. All mice phenotypically analyzed were crossed to Actin-FLP, followed by Actin Cre, and backcrossed seven generations to C57/Bl mice. B. miR-205 is developmentally regulated. Expression is evident in the branchial arches of E11.5 embryos and by E15.5, embryos demonstrate high expression in skin.

While miR-205 expression has been described in epithelial tissues, a systemic view of its expression has not been studied previously [12], [13]. Here we analyze the spatial and temporal expression of miR-205 during mouse embryonic development using an integrated LacZ reporter allele (Fig 1A) [21]. To prevent possible interference from the neomycin cassette, targeted (lacZ-neo-lox) and beta-Actin-Cre mice were crossed to generate lacZ-KO/+ heterozygous mice (Fig 1A). Mouse embryos were analyzed at several different time points, where lacZ-KO/+ and wild type mice were stained with X-Gal to determine lacZ activity. Positive lacZ activity was observed in the branchial arches and the limb buds in E11.5 and E12.5 embryos with more intense staining in the older embryos. In E12.5 embryos, there was additional lacZ activity in distinct regions of the skin. At successive developmental time points (E14.5 and E15.5), strong lacZ activity was detected in developing hair follicles and throughout the skin with more intense staining in the craniofacial and abdominal areas (Fig 1B).

### miR-205 marks epithelial cell populations in internal organs

**Figure 2:**
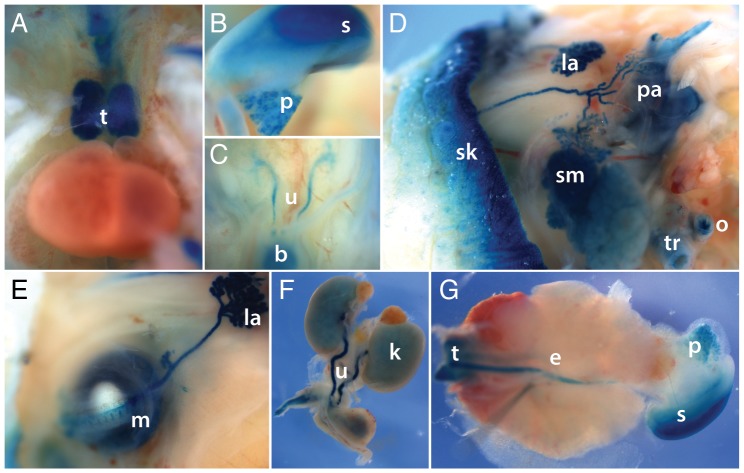
miR-205 is expressed in squamous stratified epithelium derived organs. X-gal staining of E14.5 revealed expression in the thymus [t] (**A**), stomach [s] and pancreas [p] (**B**), ureters [u] and bladder [b] (**C**). In the E18.5 embryo head, expression was salient in the several cranial organs, including the skin [sk], lacrimal glands [la], parotid glands [pa], and salivary glands [sm]. Staining was also detected in the oesophagus [o] and trachea [tr] (**D**). In addition, staining was found in the meibomian glands [m] of the eyelid (**E**). Staining was maintained in the oesophagus of the E18.5 embryo (**F**). LacZ activity is found in the ureters [u] and faint staining was also detected in the kidneys [k] (**G**).

To identify miR-205 expression in developing organs, E14.5 lacZ-KO/+ whole mount embryos were analyzed, and lacZ activity was observed in the thymus, stomach, pancreas, ureters and bladder (Fig 2 A-C). Although expression of miR-205 has been described in epithelial tissues, this is the first evidence showing expression of miR-205 in the stomach and pancreas in mice. Expression in the other tissues corroborates previous reports [22], [23]. For E18.5 stage embryos, analysis of internal organs revealed strong lacZ activity in the same organs as E14.5 embryos. Removal of the skin from the head revealed exquisite staining of the tear ducts, both the exorbital and intraorbital lacrimal glands, and the parotid and submandibular salivary glands (Fig 2D). Closer examination also revealed lacZ activity in the meibomian glands (Fig 2E). Expression of miR-205 in many of these tissues has not been previously described. Expression was observed in the esophagus, ureters, and modest lacZ activity in the kidneys (Fig 2F-G). LacZ activity was not observed in the spleen, heart, liver, or brain, corroborating other studies in mice (unpublished data) [13]. Altogether these data reveal a fine granularity of miR-205 expression profile in a broad set of squamous epithelial tissues.

### LacZ reporter provides high-resolution localization of miR-205

**Figure 3:**
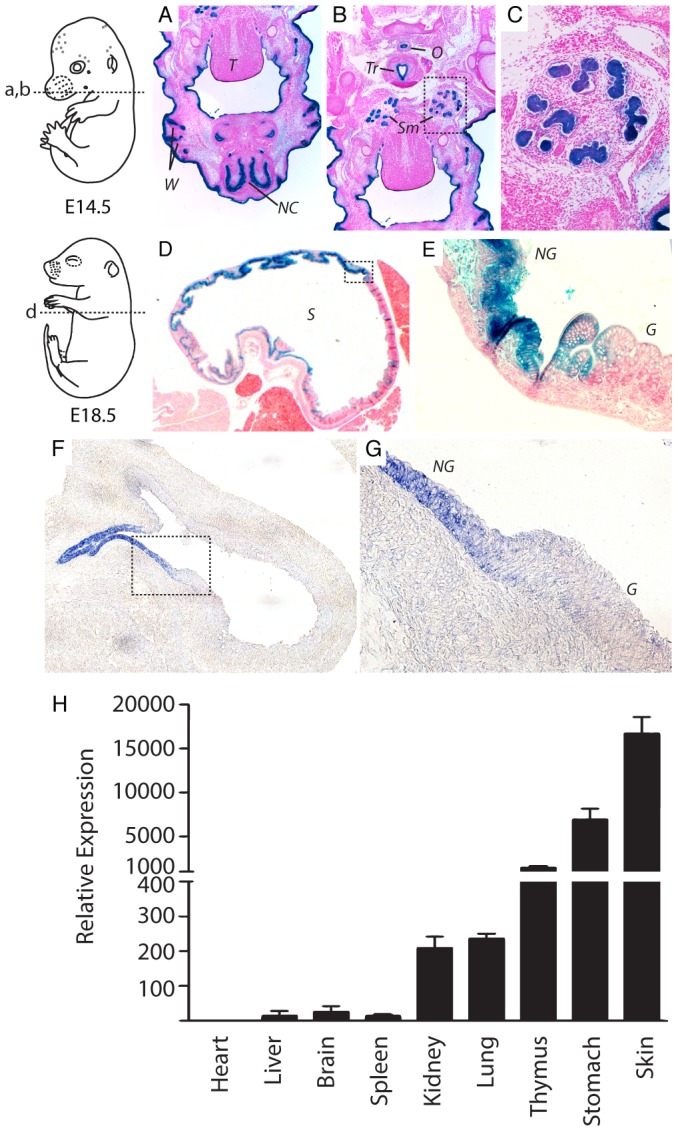
LacZ Reporter provides high resolution and accurate readout of mature miR-205. Section regions and developmental stage are indicated in the schematics on the left. (**A-C**) Transverse sections of E14.5 reveal defined expression in several cranial tissues. (**D-E**) E18.5 sectioning confirms maintained expression in tissues and refined and restricted expression in the stomach. Images in C and E are higher magnifications of the dashed box shown in B and D. (**F-G**) miR-205 LNA *in situs* on stomach sections from wild type E14.5 embryos. (**H**) Expression of miR-205 assayed by qRT-PCR of total RNA from different organs of wild type E18.5 embryos. Sno202 was used as a loading control. The error bars shown are S.E.M. Tongue [T], nasal cavity [NC], whisker follicles [W], and salivary glands [Sm], oesophagus [O], trachea [Tr], stomach [S], glandular region of the stomach [G], non-glandular region of the stomach [NG]

To gain a finer resolution on miR-205 expression, whole mount X-gal staining of embryos was followed with paraffin embedded sectioning of selected tissues. Transverse sections of X-gal-stained E14.5 LacZ-KO/+ embryos showed definitive staining in the skin, particularly in the cranial region (Fig 3A). Intense lacZ activity was also evident in epithelial cells in the nasal and oral cavities, and in developing whisker hair follicles (Fig 3A). In addition, highly specific lacZ activity was observed in the submandibular glands at high resolution (Fig 3B, C). LacZ activity in the trachea and oesophagus, specifically in the epithelium lining of their lumens, supports data from miRNA expression analyses in mouse and human (Fig 3B) [24], [25].

Analysis of E18.5 lung revealed a faint lacZ activity in the epithelial cells of bronchioles, suggestive of intermediate levels of miR-205 expression in bronchioles compared to the other lacZ-positive tissues (unpublished data). Given that mir-205 expression has not been previously reported in the stomach and that there appears to be a strong and differential pattern of lacZ activity in this organ (Fig 2B), stomach sections were examined from X-gal-stained E18.5 LacZ-KO/+ embryos. The proximal portion of the stomach is non-glandular and responsible for food storage, while the distal glandular stomach secretes enzymes essential for digestion. Distinct transcriptional networks control these two distinct parts of the stomach. The proximal stomach is composed of stratified squamous epithelium; consistent with the specificity of miR-205, lacZ activity was constrained to this non-glandular portion of the stomach (Fig 3D, E). Notably expression abates abruptly at the non-glandular and glandular epithelial junction that lines the stomach (Figure 3E). These results further emphasize the refined expression of miR-205 to stratified squamous epithelium and its derivatives.

### LacZ activity is a robust reporter of mature miR-205 expression

Some miRNAs are post-transcriptionally regulated by the inhibition of processing of the primary transcript or the miRNA precursor into the mature miRNA [26], [27]. Since lacZ activity was used as an indicator of mature miRNA expression, correlation with endogenous mature miRNA expression was investigated. In these experiments, LNA-based *in situ* hybridization and qRT-PCR was performed to compare endogenous miR-205 expression from wild-type mice with the lacZ reporter expression data. *In situ* hybridization on gut sections from wild-type embryos show that miR-205 is expressed specifically in the non-glandular epithelial cells of the stomach (Fig 3F, G), complementing the lacZ expression data. Expression was also validated by *in situ* hybridization for a limited number of other tissues (unpublished data). Moreover, total RNA from selected E18.5 wild-type embryos tissues was subjected to qRT-PCR analysis for quantitating mature miR-205 levels (Fig 3H). These data show an excellent correlation between lacZ activity and miR-205 levels, with abundant miR-205 expression in the skin, stomach and thymus. Lower levels of expression were detected in the kidney, correlating well with the weaker lacZ activity seen in this organ (Fig 2E). Together, these data validate the miR-205-lacZ reporter analysis and suggest that miR-205 is not substantially post-transcriptionally regulated at these developmental time points for these tissues.

### Partially penetrant lethality in miR-205 KO mice

We previously reported the generation of large numbers of miRNA KO mice using a conditional ‘knockout first’ strategy [21], where strategically placed recombination sites allow for gene deletion or replacement of the miRNA with a lacZ reporter (Fig 1A). In that study the miR-205 allele was zygotically replaced with a lacZ reporter in a mixed genetic background using a beta-Actin Cre transgene, yielding constitutive miR-205 KOs. These lines exhibited an embryonic/p1 lethal phenotype with essentially 100% penetrance. In the current study, the published line has been backcrossed seven generations into a C57BL/6 background, and the residual lacZ reporter ablated using consecutive Flp and Cre recombinase crosses. Although the lacZ reporter is useful for monitoring miRNA transcription activity, the transgene represents a significant DNA footprint at the endogenous locus. Function-based analysis often benefits from a clean genetic background and a cleaner ablation of the miRNA, therefore miR-205 KO analysis was performed in C57BL/6 mice having only a single frt and loxP site footprint (Fig 1A).

**Figure 4:**
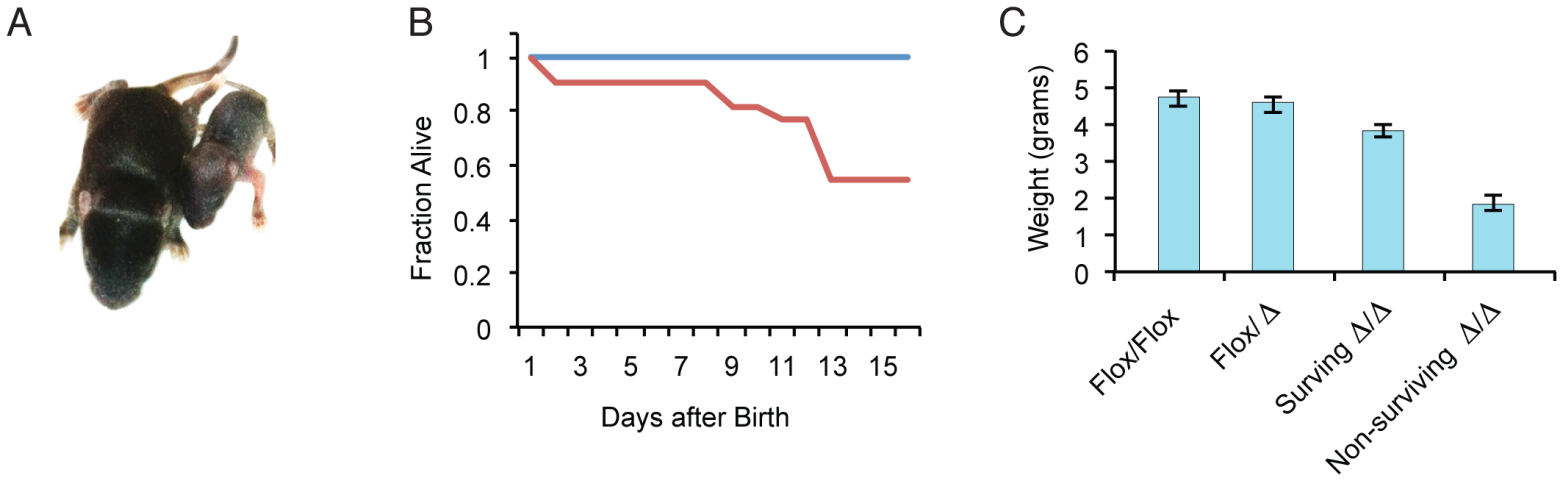
miR-205 knockout mice demonstrate postnatal lethality. (**A**) Image of WT (flox/flox, left) and KO (d/d, right) demonstrating striking differences at P10. Clear skin defects are observed with a gross decrease in the overall size of the KO. (**B**) KO mice are born normally but about 50% die before two weeks after birth (blue, flox/flox n = 20, red, d/d, n = 22). (**C**) Non-surviving KOs weigh significantly less than littermate controls at P7, averaging about half the weight of littermates. Surviving KOs show minor decrease in weight at P7 compared to littermate controls (flox/flox, n = 13, flox/d, n = 26, surviving d/d, n = 13, non-surviving d/d, n = 12), standard error reported.

Evaluation of miR-205 KOs demonstrates no apparent difference at birth between KO mice and wildtype littermate controls. Heterozygous mice appeared phenotypically identical to wild-type littermate controls. Defects become apparent within the first two weeks of birth (Fig 4A), where approximately 50% of the KO mice exhibit perinatal lethality (Fig 4B), with the remainder of the animals surviving at least 16 weeks. Although the contributing factor responsible for the observed partial penetrance is unknown, it seems likely to be an environmental variable. That said, formally we cannot rule out an epigenetic or imprinted modifier of miR-205 activity. Similarly, the current data hint that potential miR-205 genetic modifiers may be present, which could explain why the earlier report showed an earlier lethality [21]. In the earlier report, the presence of a 129 genetic background and a lacZ transgene that might affect potential enhancers present at the locus that could sensitize the viability phenotype. In any case, the miR-205 KO animals are readily distinguished by weight between p4 and p6, with non-surviving KO animals weighing half that of their littermates by p7 (Fig 4C).

miR-205 KOs also show signs of irregular skin development, evident by the appearance of dry skin and a much thinner skin layer (Fig 4A). KO mice that survive past p8 eventually develop hair, indicating that early follicle development is intact. The ability to produce mature hair follicles suggests that miR-205 plays a role in epidermal development after its differentiation from the multipotent progenitor population. Moreover, it remains a possibility that miR-205 may play an important role in the maintenance and regeneration of hair follicles. Toluidine blue dye experiments to evaluate potential skin barrier defects did not reveal any epidermal compromise at either E18.5 or P1 (data not shown). Future characterizations of skin development could reveal mechanistic underpinnings behind the histological abnormalities, and whether these phenotypes contribute to the observed postnatal lethality.

Other internal organs characterized to exhibit miR-205 expression appear morphologically normal in the non-surviving KOs, suggesting that miR-205 is not required for the gross development of these organ systems. However, the roles of miR-205 in organ function require further evaluation. Given the knockout runted phenotype and broad expression of miR-205 in several digestive organs, the possibility remains that digestive processes are being disrupted. Further studies may reveal whether digestive disorders contribute to the observed lethality and a deeper understanding of miR-205 role(s) in homeostatic function.

This study describes a single miRNA knockout that exhibits severe postnatal defects that appear within two weeks of birth. Most published miRNA knockouts lack such severe phenotypes, and those with a lethality often die embryonically or soon after birth [21]. These data support a role for miR-205 in squamous epithelium derived organs, suggested by a bevy of work aimed at delineating the targets and pathways affected by miR-205 activity. It is well established that miR-205 actively regulates ZEB1 and ZEB2, factors that ensure proper maintenance of the epithelial fate [17] and it is tempting to speculate that miR-205 plays roles in proliferation or cell migration. Improper regulation of miR-205 has been associated with increased metastasis in several tissue types, supporting a role of miR-205 in actively preventing EMT [28], [29], [30]. In addition, miR-205 has been shown to regulate proliferation and is regulated by p63, which marks epithelial stem cells, suggestive a role of miR-205 in proper maintenance of the stem cell populations in the epithelium [18], [31]. These published targets highlight a subset of highly significant miR-205 targets whose deregulation would be expected to make a profound impact in normal epithelial cell function. While all organs exhibit grossly normal development in the miR-205 KOs, it is plausible that miR-205 is active in several processes essential for proper function of these organs such in basic epithelial function or epithelial regeneration and maintenance. This report emphasizes a requirement for miRNAs in proper physiology and expands on the small list of miRNAs essential for proper development *in vivo*.

## Methods

### Ethics Statement

All mouse handling was performed according to the protocols approved by the Animal Care and Use Committee of University of California at San Francisco.

### X-Gal staining

For embryo staining, lacZ-miR-205KO/+ embryos were dissected and fixed in 4% paraformaldehyde and 0.2% glutaraldehyde in PBS for either 1 or 4 hours depending on their age. For E18.5 embryos, internal organs and brain were dissected out for better fixation and permeabilized in 0.02% NP40, 0.01% sodium deoxycholate, and 2 mM MgCl_2_ in PBS for one hour before staining. X-gal staining was done overnight at room temperature. Embryos were post-fixed in 2% paraformaldehyde in PBS and subsequently stored in 70% ethanol.

### Real-time quantitative RT-PCR (qRT-PCR)

Total RNA was purified from dissected E18.5 embryonic tissues using TRIzol reagent (Invitrogen, Life Technologies) according to the manufacturer's instructions. Reverse transcription was done using a TaqMan miRNA Reverse Transcription Kit (Applied Biosystems, Life Technologies) and Quantitative PCR was done using a Taqman miRNA assay system (Applied Biosystems, Life Technologies). Tissues from three different embryos were analyzed and the PCRs for each of these were done in triplicate and pooled for the analysis. Sno202 was used as endogenous control for normalization of the data. Expression in arbitrary units was calculated by (2^15^)×2^−ΔCT^ of miR-205 and sno202.

### *In situ* hybridization

*In situ* hybdrization was done using LNA-probes against miR-205 (Exiqon) according to the manufacturer's directions with the following modifications: Post-hybridization washes: 2xSSC at 50°C for an hour, 2xSSC at 50°C for 10 minutes, 2xSSC at RT for 10 minutes, 1xSSC at RT for 10 minutes, 0.5× SSC at RT for 10 minutes and 0.1xSSC at 50°C for 45 minutes. Blocking was done for two hours at room temperature in 0.1% goat serum. Samples were incubated 1∶5000 with AP-conjugated anti-DIG antibodies overnight at 4°C. Detection of the Alkaline Phosphatase was performed using NBT/BCIP stock solution (Roche).
